# Transposable elements: multifunctional players in the plant genome

**DOI:** 10.3389/fpls.2023.1330127

**Published:** 2024-01-04

**Authors:** Asmaa H. Hassan, Morad M. Mokhtar, Achraf El Allali

**Affiliations:** ^1^ Bioinformatics Laboratory, College of Computing, Mohammed VI Polytechnic University, Ben Guerir, Morocco; ^2^ Agricultural Genetic Engineering Research Institute, Agriculture Research Center, Giza, Egypt

**Keywords:** transposable elements, repetitive DNA sequences, TE transmission, genome diversification, satellite DNAs

## Abstract

Transposable elements (TEs) are indispensable components of eukaryotic genomes that play diverse roles in gene regulation, recombination, and environmental adaptation. Their ability to mobilize within the genome leads to gene expression and DNA structure changes. TEs serve as valuable markers for genetic and evolutionary studies and facilitate genetic mapping and phylogenetic analysis. They also provide insight into how organisms adapt to a changing environment by promoting gene rearrangements that lead to new gene combinations. These repetitive sequences significantly impact genome structure, function and evolution. This review takes a comprehensive look at TEs and their applications in biotechnology, particularly in the context of plant biology, where they are now considered “genomic gold” due to their extensive functionalities. The article addresses various aspects of TEs in plant development, including their structure, epigenetic regulation, evolutionary patterns, and their use in gene editing and plant molecular markers. The goal is to systematically understand TEs and shed light on their diverse roles in plant biology.

## 1 Introduction

Transposon elements(TEs) are mobile genetic elements that can make up a large portion of the plant and animal genome through movement processes. They can affect the genome by altering gene expression and influencing genome evolution. Some types of TEs can insert into a new location in the genome and disrupt or restore the function of neighboring genes or create new regulatory elements. Other TEs are more stable and remain in the same location in the genome for long periods of time (Kumar and Bennetzen, 1999). There are several ways to classify TEs based on their properties and behavior. One common method is based on the mechanism of movement in the genome. Based on this mechanism, there are two main classes of transposons: class I TEs and class II TEs. Class I TEs, also known as retrotransposons, are transcribed into RNA, which is reverse transcribed into DNA. The new DNA copy is able to insert itself into a new location in the genome. Class II TEs, also known as DNA transposons, can move throughout the genome by a process called “cut and paste,” in which a segment of DNA is moved from its original position and inserted into a new location (Lopes et al., 2013).

Class I TEs fall into three categories: LTR (Long Terminal Repeat), non-LTR, and DIRS (Dictyostelium discoideum TE). Each of these categories is further divided into subcategories called superfamilies. For example, the category LTR is subdivided into the superfamilies *Copia*, *Gypsy*, *Bel-Pao*, *Retrovirus*, and *ERV*. DIRS is also subdivided into *Ngaro* and *VIPER*. Non-LTR are not common in plant species and can be divided into three main groups: Long Interspersed Nuclear Elements (LINEs), Short Interspersed Nuclear Elements (SINEs), and Penelope-like Elements (PLEs) (Orozco-Arias et al., 2019). LINEs also include the R2, RTE, jockey, L1, and I superfamilies. SINEs also include the tRNA, 7SL, and 5S superfamilies. Phylogenetic studies suggest that retroviruses may have evolved from Gypsy by evolutionary processes, with the addition of a new *ENV* gene (Havecker et al., 2004; Lee and Kim, 2014). *Retroviruses* are a specific group of viruses that share similarities with retro-transposons in their genetic structure and replication mechanism. In addition, the LTR-RTs (LTR-retrotransposons) lineages have been proposed as a taxonomic category that is intermediate between the superfamily and the family and includes families with common structural and functional features and evolutionary links. In plants, six lineages belong to the Gypsy superfamily (*Athila*, *Tat*, *Galadriel*, *Reina*, *CRM/CR*, and *Del/Tekay*) and seven belong to the *Copia* superfamily (*TAR/Tork*, *Angela/Tork*, *GMR/Tork*, *Maximus/Sire*, *Ivana/Oryco*, *Ale/Retrofit*, and *Bianca*) (Wicker et al., 2007; Du et al., 2010).

Class II DNA transposons are divided into subclasses, including *Helitron*, Cut-and-Paste, *Crypton*, and *Maverick/Polinton* (Bao et al., 2010). The cut-and-paste subclass is further divided into superfamilies such as *hAT* and *Tcl/mariner*, each containing different families such as *Ac/Ds* and hobo in *hAT* and *Tc1* and mos1 in *Tcl/mariner*. *Tcl/mariner* is further categorized into families such as *TLEs*, *MLEs*, and Pogo-like elements. *MLEs* with terminal inverted repeats (TIRs) of different lengths and transposase-encoding ORFs generate target site duplications (TSDs) of different lengths upon integration (Lopes et al., 2013). In addition, class II DNA transposons usually consist of TIRs that encode a transposase protein responsible for facilitating their movement. In plants, the transposase protein recognizes the specific TIR sequences and plays a critical role in classifying DNA TEs class II into several superfamilies, including *CACTA*, *hAT*, *Merlin*, *Mutator*, *P-element*, *PIF*, *PiggyBac*, and *Tc1/Mariner*(Wicker et al., 2007).

**Figure 1:**
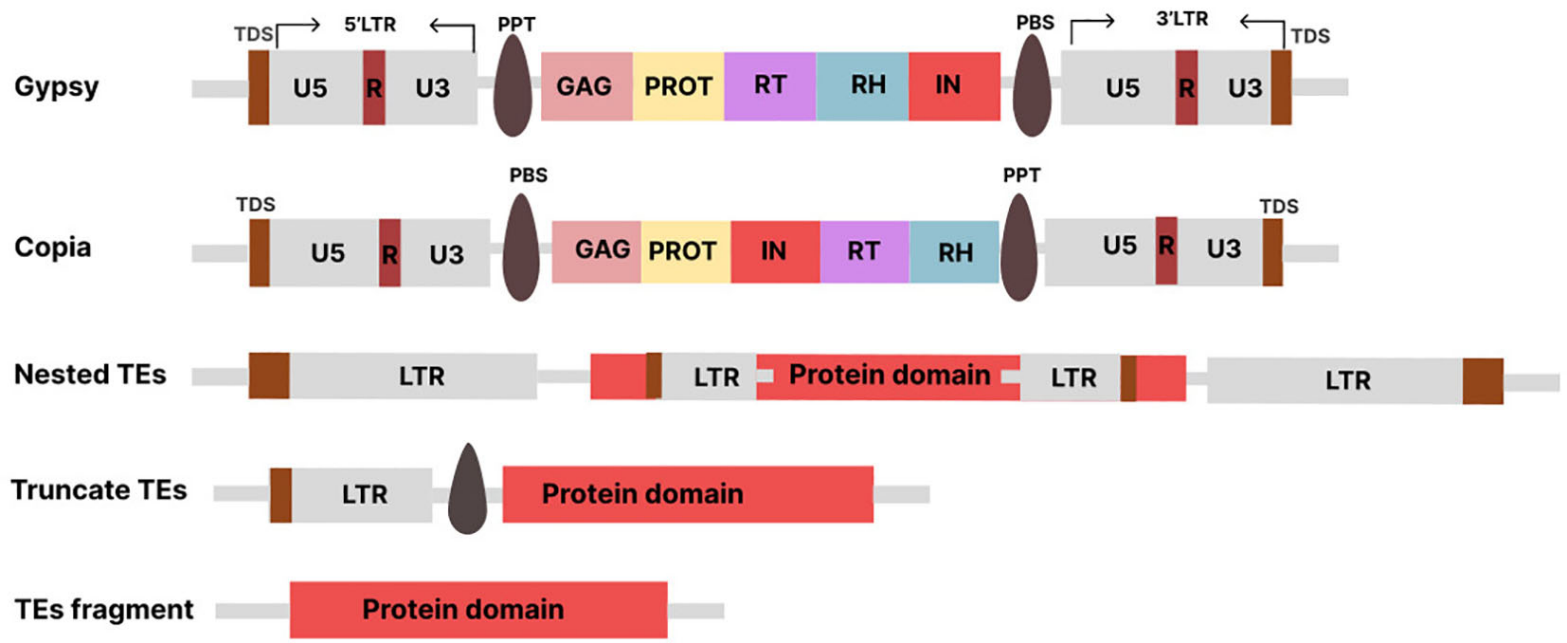
Various TE types: Conserved structures of *Copia*, *Gypsy*. Nested and Fragments LTR-RTs (Truncate TEs, TEs fragment). The structures are not drawn to scale.

Depending on the elements’ structure, the LTR-RTs may be active or inactive in the genome. The active elements may be putative autonomous or non-autonomous depending on how complete the LTR-RT structure is. The autonomous elements are referred to as LTR-RTs, which can move independently within the genome because they contain all the protein-coding domains necessary for their mobilization. On the other hand, the non-autonomous LTR-RTs, are the elements that lack one or more of the protein-coding domains required for mobilization. The complete structure of LTR-RT consists of two identical LTRs surrounded by TSDs of typically 4-6 base pairs (bp), a PBS (primer binding site), a PPT (polypurine tract), a *Gag* gene encoding a polyprotein, and a *Pol* gene (Mokhtar et al., 2023). The *Pol* gene encodes domains such as reverse transcriptase (RT), RH (RNase H), IN (integrase), and PR (protease). Some LTR-RTs also have a *ENV* -like (envelope) protein, similar to retroviruses. In plant genomes, the assignment of LTR-RTs to *Copia* and *Gypsy* is based on the order of occurrence of RT and IN within the *Pol* region (Ma et al., 2019). The structure of LTR consists of three functional regions known as U3, R, and U5 (**Figure 1**). The U3 is located upstream of the transcription start site (TSS) and contains the promoter and regulatory motifs involved in transcriptional regulation. The U5 is located downstream of the transcription termination site (TTS) in the 3’LTR. The R region is located between the U3 and U5 regions. The 3’LTR contains regulatory sequences and a functional promoter that can affect the expression of neighboring genes. The U3 region is highly variable, which affects the expression of LTR-RTs and their responses to stress-related signaling molecules. Different species and subfamilies of LTR-RTs exhibit variations in their U3 regions, resulting in different regulatory motifs and stress responses (Grandbastien, 2015). The LTR is reconstituted after reverse transcription, resulting in two identical LTRs. The divergence between the two LTRs of the same LTR-RT is used to estimate the insertion time. LTR-RTs, such as BARE in barley, use different promoters to generate non-polyadenylated RNA templates and polyadenylated mRNA transcripts.

In some cases, retrotransposition involves the insertion of a complete LTR-RT element at a new location in the genome. Successful insertion of LTR-RT results in intact or nested LTR-RTs (**Figure 1**). However, the process can sometimes fail, resulting in the deletion of one of its structural sequences and the reformation of LTR-RT, including truncated and solo-LTR-RTs. Intact LTR-RTs are complete LTR-RTs that have retained their full genetic structure and are able to mobilize in the genome using an RNA intermediate. Nested LTR-RTs refer to a situation in which one LTR-RT is inserted into another LTR-RT, resulting in a nested arrangement. Removal of the internal region of the intact LTR-RT results in a solo-LTR-RT and is used to indicate the rate and effectiveness of removal of LTR within a genome (Ma et al., 2004). The truncated LTR-RTs are retrotransposons in which one or both LTRs have been partially deleted or truncated. The truncated LTR-RTs may also result from illegitimate recombination processes, leading to deletions and translocations. Fragments that share similarities with incomplete retrotransposon sequences with no discernible structural features are referred to as remnants (Grandbastien, 2015). Evidence suggests that the inactive or nonfunctional LTR-RT elements within the *Gypsy* and *Copia* superfamilies can be mobilized by active elements from the same subfamily, leading to an additional level of parasitism phenomenon termed “hyperparasitism” (Wicker et al., 2007; Du et al., 2010). Each class of transposons has corresponding nonautonomous forms that lack one or more sequences necessary for transposition. For example, class I TEs include TRIM (terminal repeats in miniature; (Witte et al., 2001)), LARD (large retrotransposon derivatives; (Kalendar et al., 2004)), TR-GAG (terminal repeat retrotransposons with *GAG* domain; (Chaparro et al., 2015)), BARE (barley retroelement; (Suoniemi et al., 1996)), SINEs, and Morgane elements. The form of non-autonomous transposons of class II is called MITE (Miniature Inverted-repeat Transposable Elements).

TEs exhibit both vertical and horizontal transmission, with LTR-RTs usually inherited vertically, but there are rare cases of horizontal transmission, as observed in the case of the non-LTR-RT *AdLINE3*. Flanking sequences of TEs influence their preferred insertion sites, which are often located in regions actively involved in gene expression, as seen for *Mu1* and *Spm* in maize. LTR-RTs have low DNA copy numbers in the genome, and many are associated with DNA methylation, especially in heterochromatin regions, as seen in *Beta vulgaris*. In addition, LTR-RTs contribute to plant genome diversification, as reported in a study by Park et al. (2021) on genomes of *Vitis* species (Cresse et al., 1995; Bennetzen, 2000; Zakrzewski et al., 2017; Park et al., 2021; Ramakrishnan et al., 2023).

**Figure 2:**
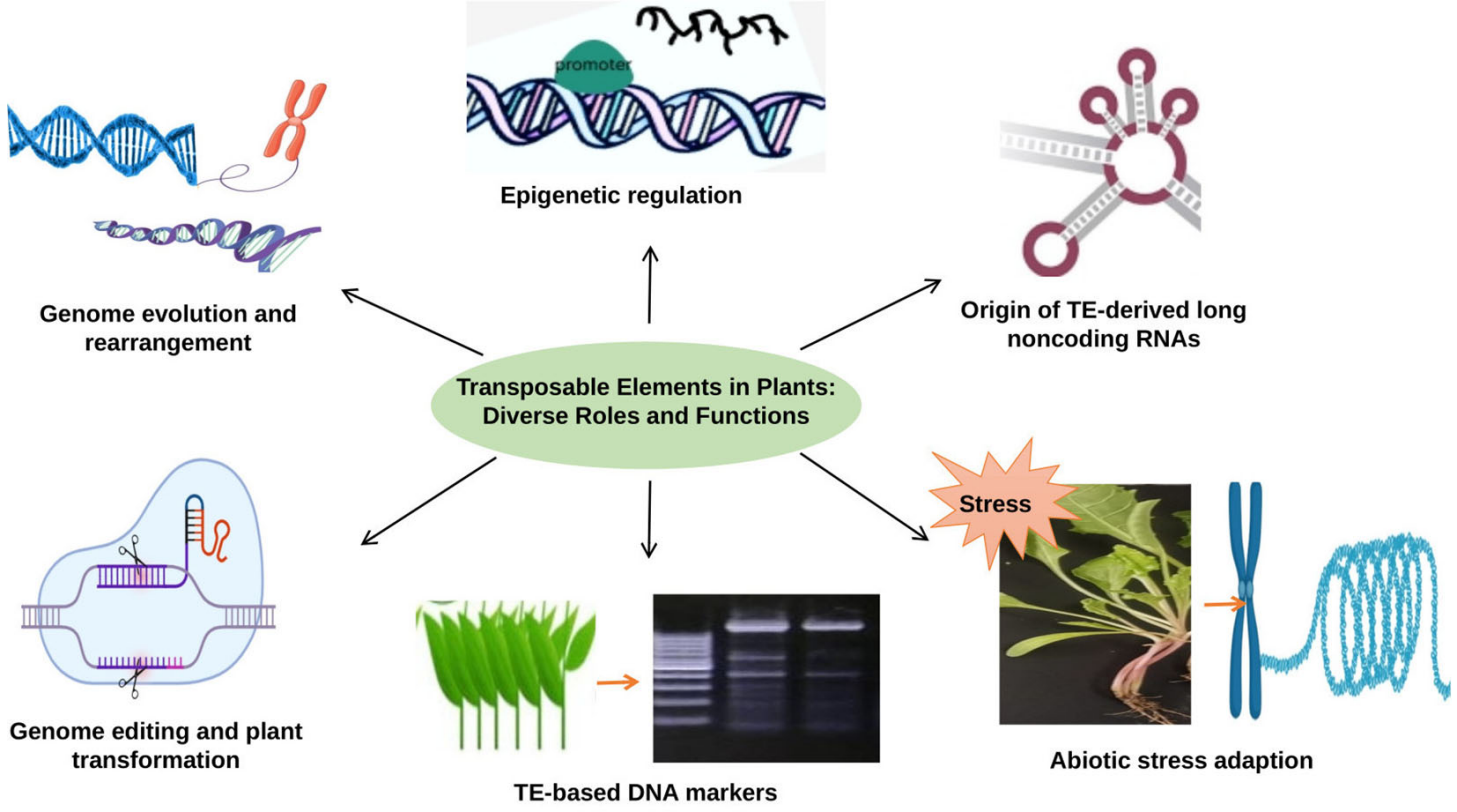
The role of TEs in plant genomes: TEs are involved in epigenetic regulation, genome evolution and rearrangement, abiotic stress adaption, generation noncoding RNA, genome editing and used as a molecular marker.

TEs are versatile genetic elements that play diverse and essential roles in plant genome function and evolution. TEs have the ability to influence gene regulation through epigenetic modifications and play a key role in shaping the expression patterns of neighboring genes. They also contribute to the formation of non-coding RNAs, such as long non-coding RNAs (lncRNAs), which have been shown to be crucial regulators of various physiological processes in plants. In addition, TEs profoundly impact genome evolution and rearrangement, driving genetic diversity and speciation. These elements have also been used for genome editing and transformed plant production, enabling precise and targeted modification of plant genomes. In addition, TEs are associated with adaptation to abiotic stress, as they can be induced in response to environmental stress and play an important role in transcription that responds to stress. Their sequences are widely used as molecular markers to study genetic variation and diversity among different plant species (**Figure 2**). In this review, we highlight the roles of TEs in plant genomes that offer a wealth of functions and applications that have revolutionized our understanding of plant genetics and biotechnology.

## 2 SatDNAs formation from TEs and their impact

**Figure 3:**
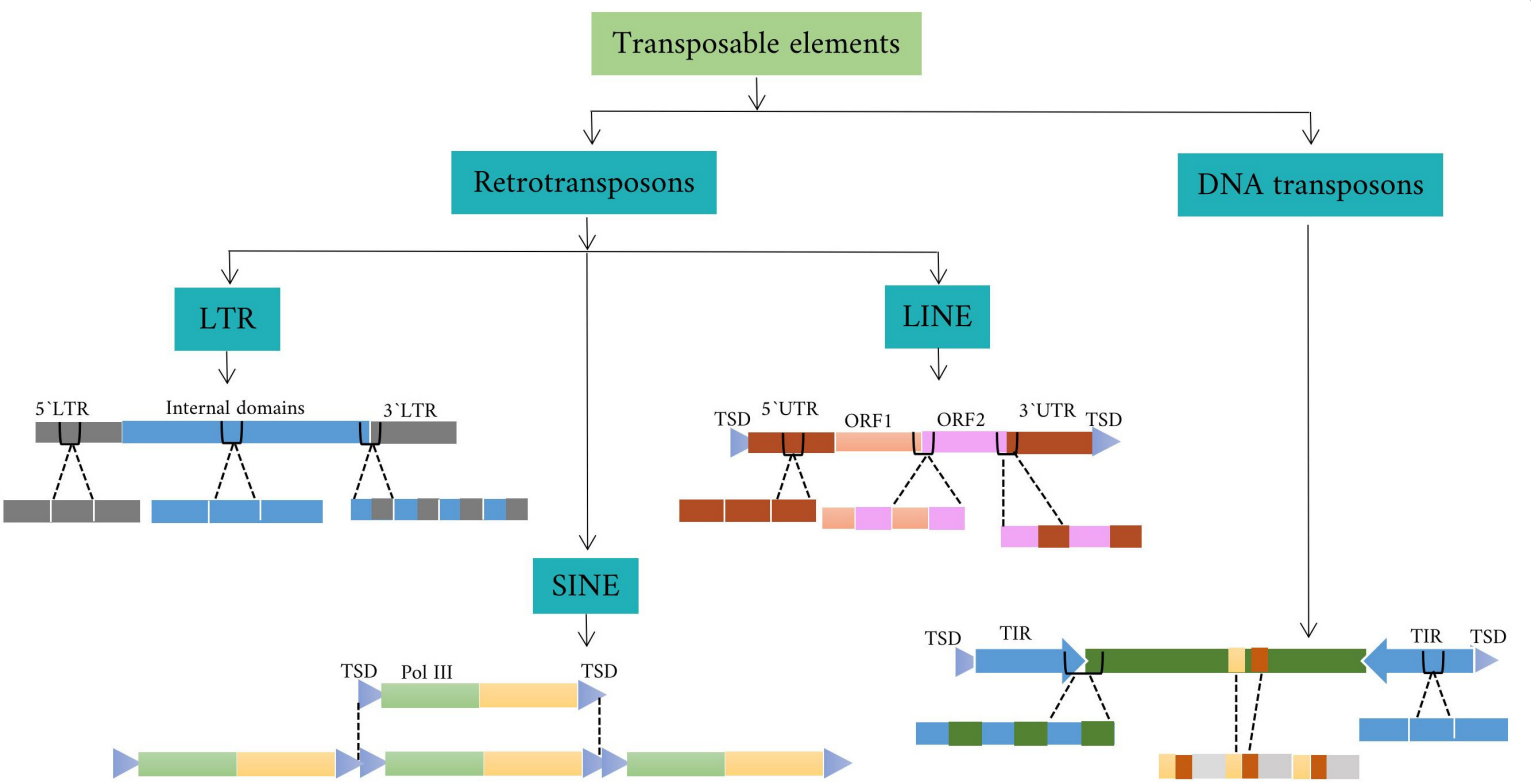
satDNAs are formed by the amplification of fragments of TEs and consist of repeating units known as monomers. The potential origins of these satDNA monomers are mapped onto simplified diagrams of various TE types, including retrotransposons(LTR, LINEs, and SINEs), and DNA transposons.

Satellite DNAs (satDNAs) and TEs are an important class of repetitive DNA that are highly diverse and often part of eukaryotic genomes. They play an essential role in genome structure, function, and evolution. SatDNAs are tandemly repeated sequences that are often localized in specific regions of the genome, such as centromeres and heterochromatin. satDNAs typically consist of longer repeat units, ranging from hundreds to thousands of base pairs in length. SatDNAs are involved in centromere function, chromosome segregation, and maintenance of genome stability. Heterochromatin is an inactive region of the genome that serves as a prominent convergence zone for both TEs and satDNAs in eukaryotic genomes, particularly in (peri)centromeric regions (Heslop-Harrison and Schwarzacher, 2013). Plant centromeres exhibit extensive mixing of satDNAs and TEs, particularly the CR family of *Gypsy* superfamily found in several grass species such as rice (Cheng et al., 2002) and wheat (Liu et al., 2008). These *CR* elements not only associate with centromere-specific satDNAs but also interact with the centromere protein *CenH3*, suggesting that they are actively involved in the function of grass centromeres. In addition, recent studies on the centromeres of maize and wheat suggest that CR elements play a leading role in the formation of the kinetochore and can repress satellite sequences (Wolfgruber et al., 2009; Li et al., 2014). In *Arabidopsis thaliana*, introduction of the centromeric LTR-RT *Tal1* from a related species, *Arabidopsis lyrata*, by transformation shows a preference for targeting the specific centromeric satDNAs repeats of *A. thaliana*, despite their distinct differences (Tsukahara et al., 2012). Plant *CRM* retrotransposons could specifically target centromeres *via* a putative domain in their integrase. SatDNAs and TEs are predominantly located in heterochromatic regions, possibly due to gene-poor domains that allow safe propagation. Nevertheless, there is evidence that they are actively involved in centromere structure and function, suggesting an interaction between satDNAs and TEs. This interaction leads to simultaneous amplification and the formation of new sequences that integrate and/or combine the building blocks of both satDNAs and TEs (Neumann et al., 2011) (**Figure 3**).

satDNAs may indeed derive from mobile elements, especially transposons and retrotransposons, as shown by sequence homology. These mobile elements could contribute to the formation and spread of satDNAs in the genome. Longer satDNAs monomers, typically consisting of more than 500 bps, often originate from specific regions of retrotransposons, such as the LTRs and UTRs (untranslated regions). In these cases, satDNAs may be predominantly located in (peri)centromeric regions of the genome. These regions are associated with the centromere, the chromosomal region responsible for proper chromosome segregation during cell division. This is in contrast to “classical” satDNAs, which can be scattered throughout the genome. An example of this phenomenon is found in maize and potato, where in maize two pericentromeric satDNAs originate exclusively from the LTR and UTR regions of centromeric retrotransposons and belong to the *CRM1* and *CRM4* subfamilies (Sharma and Nandineni, 2014). This highlights how retrotransposons can serve as a source of satDNAs and contribute to the formation and organization of repetitive elements in specific genomic regions.

satDNAs can arise from different types of TEs and their structural components (**Figure 3**). In this case, some regularities in the segments of TEs are observed in the amplified tandem repeats, indicating a directed transition from TEs to satDNAs, although a reverse process is also possible (Kejnovsky et al., 2006; Macas et al., 2009). Moreover, the exact pathway of satDNA formation from TEs is not fully understood, but it involves structural elements such as palindromes, direct and inverted repeats, and stem-loop structures that play a role in the mobilization of TEs and the formation of tandem repeats. Furthermore, several mechanisms, such as illegitimate recombination and deletions triggered by double-strand breaks and excisions, likely contribute to TE rearrangements leading to the formation of sequence segments that can subsequently be amplified into tandems to form satDNA arrays (Meštrović et al., 2015). In addition to other mechanisms such as integrating an entire retrotransposon, fusion of different truncated TEs, or combining TEs with other genomic sequences. An example is the potato St3-294 satDNA, whose monomer length is more than 5.4 kb and originates from the nonautonomous LTR-RT located subtelomerically on chromosome 9 (Gong et al., 2012). In addition to retrotransposons, the formation of satDNA can also be affected by DNA transposons; TIR sequences, in particular, can change the structure of satDNA. In *Arabidopsis thaliana*, two satellites, *Ensat1* and *Ensat2*, were formed based on the TIRs of the DNA transposon *Atenspm2* (Kapitonov and Jurka, 1999). The complex monomer of *Ensat1* has similarity to a 499 bps of *Atenspm2*, whereas a segment of *Atenspm2* of about 151 bps is present in the *Ensat2* monomer. In addition, the remaining portion of the *Ensat2* monomer has 85% similarity to the internal portion of another transposon called *Arnold1* (Langdon et al., 2000).

Tandem repeats are also commonly found in TEs, with DNA transposons having a higher frequency of tandem repeats than retroelements. TEs *Tnat1* and *Tnat2* in *Arabidopsis thaliana* contain central tandem repeats of either 60 or 240 bps in length (Noma and Ohtsubo, 2000). On the other hand, foldback transposons in *Oryza sativa* have been reported to have short tandem repeats of less than 90 bp within their TIR structures (Cheng et al., 2002). Analysis of whole-genome sequencing data revealed that internal tandem repeats of TEs may be present in the form of satDNAs arrays. In the 3’ UTRs of the *Tat* lineage, which is one of the LTR-RTs in several plant genomes, there are frequent occurrences of variable tandem repeats. These internal tandem repeats can form satDNAs that are present in several plant species such as *Pisum sativum*, *Hordeum vulgare*, and *Glycine* sp (Makarevitch et al., 2015). Understanding the origin and evolution of satDNAs from TEs provides valuable insights into the dynamics of genome organization and the role of TEs in shaping genome structure and function (Makarevitch et al., 2015).

## 3 TEs in plant genome evolution and speciation

Genome evolution involves chromosomal rearrangements, duplications, and genetic variations. TEs play a critical role in shaping genome evolution by facilitating recombination and allowing genes to move to other locations. The interaction between transposons and plant genomes has enabled adaptations to unfavorable environments as well as processes such as hybridization and polyploidy. Large genomes can arise from small genomes by one or more amplification mechanisms. However, the rate of DNA loss in small genomes is greater than that of TE amplification for DNA gain, as shown in cotton and its families (Canapa et al., 2016). The frequency of certain TE families differs among species and even within closely related species. Some TE families have elevated copy numbers within specific lineages. Examples include a single type of LTR-RT that dominates the genome of *Capsicum annuum* and a specific retrotransposon that makes up a significant portion of the genome of *Hungarian vetch* (Neumann et al., 2006).

TEs have a fascinating influence on plant development, especially on the phenomenon of variegation in flowers. Variegation in plants is characterized by unpredictable expression of anthocyanins, resulting in the formation of spots or sectors of different color on flowers. This variability can be attributed to the movement of TEs within genes associated with anthocyanin synthesis. When TEs insert themselves into these genes, they can disrupt their normal function, resulting in variegated flower phenotypes. In addition, TEs are able to regulate gene expression by inserting themselves into promoters or other regulatory sequences, resulting in altered tissue-specific expression patterns. For example, TE insertions into genes such as *CHS* and *DFR* were found to result in different color sectors in petals. These observations highlight the important role of TEs in shaping diverse and visually striking floral phenotypes (Jung et al., 2019). In addition, TEs have practical applications in biotechnology to generate new flower genotypes. They are used in breeding techniques such as insertional mutagenesis and transposon tagging, allowing researchers to study gene function and regulatory elements effectively. In particular, the presence of TEs in genes involved in the regulation or biosynthesis of anthocyanins affects flower coloration in several species, including *Nicotiana tabacum*, *Ipomoea* sp. (Pandita and Pandita, 2023), and *Petunia* sp. The dynamic interaction between TEs and plant genomes reveals their remarkable contribution to the fascinating diversity of flower colors and phenotypes. Understanding the influence of TEs on flower development expands our knowledge of plant genetics and offers exciting opportunities for biotechnological advances in breeding and genetic studies.

TEs have the ability to promote recombination between specific genomic regions, which can lead to chromosome rearrangements. Studies in *Arabidopsis thaliana* have revealed the molecular basis of epigenetic inheritance and its complex interaction with regulatory networks such as DNA methylation, RNA interference, and histone modification. Specific transposon mutations have been found to affect these processes, resulting in variations in response and inheritance patterns. In addition, chromosome rearrangements caused by transposons can affect gene expression by altering the regulatory sequences of neighboring genes. As reported, minor gene expression changes may occur when its coding region is affected by the regulatory sequence of another gene. For example, variations in the expression of epigenetic modifiers involved in downstream targets have been associated with decreased gene expression in *A. thaliana* related to TEs activation and methylation (Slotkin et al., 2009).

TEs play a critical role in rates of speciation and divergence, but their functional significance at the molecular level in the origin of new species is still unclear and unexplored. TEs have the remarkable ability to amplify DNA sequences and serve as drivers of speciation in organisms. Their impact on genome evolution is substantial, with two main mechanisms contributing to DNA sequence amplification: replicative transposition and recombination. In recombination events, TEs located at different positions on a chromosome can cause cleavage of the intervening DNA. This results in the deletion of the intervening sequence on one strand and its duplication on the other. This process is responsible for the amplification of short interspersed sequences and processed pseudogenes in eukaryotic genomes. Moreover, retrotransposon elements are restricted by site-specific duplications and inserted at new sites after reverse transcription of RNA. TEs play a critical role in shaping rates of speciation and divergence in organisms. By promoting genetic variation and facilitating genomic rearrangements, these TEs contribute to the formation of new species. However, the precise molecular mechanisms and functional significance of TEs in the origin of new species are still the subject of ongoing research and exploration (Bennetzen and Wang, 2014). The interplay of TEs and speciation is a fascinating area of research that has the potential to provide deep insights into the genetic processes underlying the origin of new species. Understanding the role of TEs in genome evolution and speciation can deepen our knowledge of the diversity and complexity of life forms on our planet.

Polyploidy, a widespread phenomenon in plants, plays a central role in speciation by duplicating entire genomes and leading to extensive genetic repeats. There are two main types of polyploids: Autopolyploids, in which multiple copies of the same ancestral species are present, and allopolyploids, which result from the fusion of genomes from different species. TEs have their own promoters and enhancers, allowing them to regulate gene expression and create new regulatory networks. TEs play a key role in genome restructuring, gene loss, and maintenance of genomic balance after duplication events. Allopolyploidization can lead to TE transcription and activation, it can also lead to an increase in TE copy number in diploid species, as well as an increase in proliferation, which is observed after polyploidization. The impact of TEs on a genome is influenced by the parental species and the specific TE families. Polyploidization does not always result in increased TE copy number, and different TE families may experience different regulation within a genome. TE Regulatory mechanisms vary between TE families and genomes, with factors such as TE type, copy number, and promoter arrangement. Both polyploidy and amplification of LTR-RTs contribute to the expansion and evolution of plant genomes (Wood et al., 2009; Parisod and Senerchia, 2012). Polyploidization processes can activate specific groups of TEs, whether autopolyploidization or allopolyploidization processes. In addition, increased retention of TE insertions in polyploids contributes to TE copy number expansion due to gene duplication. In wheat, epigenetic silencing of TEs can reduce the expression of nearby genes, a phenomenon associated with TE silencing during polyploidization, causing alterations in gene expression. Following polyploidization, genes located near reactivated TEs may be influenced by various TEs, leading to changes in their transcriptional activity and chromatin rearrangement. Reactivated TEs also have the capacity to duplicate themselves, inducing further transcriptional modifications in adjacent genes. For polyploids, a diminished control in TE silencing post-polyploidization contributes to the restoration of genome functionality, as observed in studies (Kashkush et al., 2003).

The study of polyploidy and TE regulation provides valuable insights into the genetic mechanisms that drive plant genome evolution, particularly concerning speciation and genome diversification. Understanding the interplay between polyploidy and TEs expands our knowledge of the genetic processes that determine plant species diversity. References (Kashkush et al., 2003; Wood et al., 2009; Parisod and Senerchia, 2012) are likely to provide further evidence and in-depth studies on the role of polyploidy and TEs in plant genome evolution.

## 4 TEs and epigenetics in plants

As our understanding of the biochemical processes involved became clearer and epigenetics gained popularity in the second half of the 20th century, the development of epigenetic silencing was a strategic adaptation aimed at controlling the spread of TEs and mitigating their perceived deleterious effects. TEs exhibit epigenetic features such as heterochromatin formation, DNA methylation, RNA interference (RNAi) and histone marks. These epigenetic modifications are involved in the control of various biological processes, including transgene and transposon repression, imprinting control and control of developmental processes (Ito, 2014). Specificity of DNA methylation is established by RNA-directed DNA methylation (RdDM) involving the plant RNA polymerases Pol IV and Pol V and mediated by 24-nucleotide siRNAs (Fedoroff, 2012). Our understanding of RdDM in plants is largely based on the study of the *FWA* gene in Arabidopsis. Normally, transcription of the *FWA* gene is repressed by DNA methylation of a specific fragment within the TE SINE (Short Interspersed Nuclear Element) in its promoter. When a transgenic *FWA* gene is introduced, it is efficiently silenced if an endogenous methylated copy of the gene is present (Chan et al., 2006). In plants, the RNA-based silencing pathway has evolved to include multiple paralogs of genes such as Dicer and Argonaute, each of which performs specific functions in different aspects of the silencing process (Lisch, 2009). In addition, DNA methylation exerts an influence on histone modifications, as shown by recent structural evidence demonstrating a synergistic positive feedback loop between H3K9me2 and CHG methylation for transposon silencing. This mechanism involves recruitment of the H3K9 methyltransferase Kryptonite (KYP) to methylated CHG DNA through its SET-ASSOCIATED and RING-ASSOCIATED (SRA) domains (Cui and Cao, 2014).

In an early study, changes in the methylation status of Mutator transposons in maize led to changes in the variegation patterns observed in maize kernels (Chandler and Walbot, 1986). In a maize line with a lethal phenotype characterized by the inability to produce adequate photosynthetic machinery, the expression of the phenotype was evident when an unmethylated Mutator transposon insertion was present. Conversely, the phenotype was suppressed when the transposon was methylated and rendered immobile (Martienssen et al., 1990). The reversal of mutants and the irregular phenotypes induced by TEs shed light on the observations of Barbara McClintock regarding the movement and mutations induced by activator-dissociator (Ac/Ds) transposons (McClintock, 1950). Additionally, deactivation of DNA methyltransferase 1 (MET1), the primary methyltransferase responsible for maintaining methylation of cytosines upstream of guanines (CGs), leads to activation of several families of TEs in Arabidopsis (Mirouze et al., 2009). Some Pack-MULEs exhibit features associated with active protein-coding genes, such as the presence of active histone marks, enrichment of DNase I hypersensitive sites (DHS), low DNA methylation, and high incidence of transcription and translation. This suggests that these specific Pack-MULEs have the ability to play a role in the functional aspects of the rice genome. In addition, there is evidence that Pack-MULEs may have an impact on chromosomal base composition and expression patterns in the rice genome (Zhao et al., 2018).

Hypomethylation of an intronic LINE retrotransposon in oil palm during tissue culture leads to palm mantling and an associated drastic yield reduction (Ong-Abdullah et al., 2015). When plants are exposed to environmental stress, activated TEs can jump to new genomic locations, leading to an increase in TE copy number or TE silencing, which in turn leads to changes in gene function with positive or negative effects (Quadrana et al., 2019). TE insertions within genes can inactivate them or alter their splicing patterns, leading to new proteins (Muñoz-López and García-Pérez, 2010). The insertion of TEs near genes can lead to new control mechanisms and influence gene expression and function (Chuong et al., 2017; Mokhtar et al., 2023). For example, the retrotransposon *Gret1* upstream of a gene that regulates anthocyanin biosynthesis in grapes causes a white color phenotype, while the presence of a solo-LTR at the insertion site can partially convert the white berry phenotype to a red color (Azuma and Kobayashi, 2022) and retrotransposon insertion into a MADS-box gene in primrose flowers alters the “tube-in-tube” phenotype (Li et al., 2014). Epigenetic processes, such as vernalization, also play a crucial role in regulating environment-dependent developmental transitions in plants. Vernalization, a cold requirement for germination and the transition to flowering, is an example of this phenomenon. In Arabidopsis plants, vernalization is essential for the initiation of flowering; otherwise they grow vegetatively. This process involves the cold-induced epigenetic silencing of the FLOWERING LOCUS C (*FLC*) gene, a repressor of flowering. The extent of silencing increases with the duration of the cold period and involves the production of non-coding *FLC* transcripts, resulting in histone modifications that deactivate gene transcription (Song et al., 2012).

The intricate link between transposon silencing and centromere function is illustrated by the conserved yet variable nature of centromeres. Due to the flexibility of centromere sequences, epigenetic explanations dominate the discussions (Han et al., 2006). Plant centromeres are composed of repetitive arrays and TEs flanked by pericentromeric regions rich in silenced TEs. Although there is little direct evidence for RNA-based chromatin remodeling in plant centromeres, the diversified RNAi pathway in plants and the presence of centromeric retrotransposons (CRs) in plant centromeres suggest a possible role (Zhong et al., 2002). In maize, CRs interact with CENH3, the centromere-associated histone, and in rice, CRs exhibit H3K9 methylation and siRNA targeting. This intricate interplay underscores the complexity of RNA-based mechanisms in the function of the centromere(Neumann et al., 2007).

Many TEs in plants play a crucial role in regulating the expression and function of neighboring genes. For example, mutation of *AtCOPIA4*, a retrotransposon near disease resistance genes, impairs downy mildew resistance in Arabidopsis. Another example is the heat-resistant behavior conferred by *ONSEN*, a TE in Arabidopsis, to neighboring genes. In rice, miniature TEs (Miniature Inverted Repeats, MITEs), especially Miniature Ping (mPing), have significant effects (Cui and Cao, 2014).

## 5 TEs in plant stress responses

Anthropogenic activities such as fossil fuel burning, deforestation, and land use change are the main causes of global warming, leading to an increase in greenhouse gas emissions and subsequent global warming. As a result, average temperatures are rising, precipitation patterns are changing, and extreme weather events such as droughts, floods, heat waves, cold snaps, and salinization are increasing (Edenhofer, 2015). These climatic changes pose major challenges to agriculture, as abiotic stressors severely hinder crop development and lead to significant crop losses, accounting for about 50% of the total yield losses (Rockström, 2003).

Remarkably, TEs play a central role in plant responses to abiotic stresses, which include non-living environmental factors such as drought, heat, cold, salinity, and heavy metals. TEs and the genes in their environment are thought to cause transcriptional activation under stress conditions, contributing to the plant’s adaptive response (Pereira, 2016). Stress can have different effects on TEs depending on the context. Normally, TEs are tightly regulated by epigenetic mechanisms to prevent them from being rearranged during normal development. However, stress can cause TE repression to be inhibited and reactivate them. In addition, TEs have their own promoters and regulatory sequences that allow them to affect the regulation and expression of neighboring genes in different ways. For example, studies in rice have shown that the insertion of transposable miniature inverted repeat elements (MITEs) within 100 bp of the transcription start site of a gene can be induced by salt and frost stress (Naito et al., 2009).

TEs also serve as a source of novel cis-regulatory elements for plants when inserted or transposed into the genome (Ito et al., 2016). For example, insertion of a class II TE named *Hatvine 1-rm* into the promoter region of the grapevine *VvTF1A* gene was found to upregulate its expression in both reproductive and vegetative tissues, indicating the role of TEs in somatic and reproductive cell variation. However, the relationship between TEs and abiotic stress-activated gene expression is complex, as TEs have been associated with both upregulation and downregulation of nearby genes (Stapley et al., 2015). Moreover, TEs can be initially activated and later repressed in response to stress (Secco et al., 2015).

A study examining the response to heat stress in different ecotypes revealed intriguing differences in the expression of TEs. Some ecotypes showed higher TE expression under stress, while others showed lower expression (Lapp and Hunter, 2016). These results highlight the role of TEs in regulating genes involved in abiotic stress response. In maize, for example, a transposon insertion in the promoter region of a drought tolerance gene affects its epigenetic regulation, leading to differences between maize cultivars from temperate and tropical regions. About 20% of abiotic stress-responsive genes in maize are affected by these transposons, indicating selection and evolution of stress-protective alleles in natural populations. Also in rice, insertion of the *mPing* transposon into the 5’ regions of genes leads to up-regulation of downstream genes responding to stress conditions (Naito et al., 2009; Makarevitch et al., 2015).

TE insertions may contribute to phenotypic variation and enable plants to respond rapidly to environmental stress, facilitating adaptation to new environments. A study analyzing the genomes of *Capsella rubella* and *Coreopsis grandiflora* revealed highly polymorphic TE insertions in *C. rubella*, particularly in the promoters and downstream regions, compared with the outcrossing species *C. grandiflora*. Some of these TE insertions were associated with significant changes in the expression of neighboring genes and provided variation in an otherwise inbred species (Hou et al., 2019). TEs located near or within genes can induce methylation that then spreads to neighboring genes, resulting in decreased gene expression and rapid plant response to stresses (Galindo-González et al., 2018). This response is closely related to phytohormones, particularly abscisic acid (ABA), where DNA methylation is thought to play a role in ABA-dependent gene expression (Gohlke et al., 2013). The relationship between ABA-induction and DNA methylation under drought stress was further investigated using the ABA-deficient *Z. mays* mutant vp10. Differentially methylated regions with sequence similarities to TE-elements were discovered (Sallam and Moussa, 2021).

TEs containing stress-responsive cis-regulatory elements show rapid responses to environmental stress through epigenetic modifications. For example, the *Rider* retrotransposon, which belongs to the *Copia* superfamily in the tomato plant genome, is activated upon drought stress and ABA (Sallam and Moussa, 2021). Its LTRs contain elements associated with the dehydration response and ABA signal transduction, suggesting that it is involved in the response to drought stress (Ito et al., 2016; Benoit et al., 2019). In addition, TEs such as *Tnt1* in tomato and tobacco can be specifically triggered and activated by cold temperatures, especially frost. However, the relationship between TEs and stress is diverse and complex, with different studies reaching different conclusions. TEs may be activated or repressed depending on the nature of the stress and the properties of the specific TEs, or they may become active only after being exposed to the stress. These elements can also influence the expression of stress-responsive genes by generating allelic variations and new expression patterns through their insertion into promoter regions. In addition, domesticated TE genes contribute to the expression of abiotic stress response, as shown by the exaptation of TEs in plants (Mhiri et al., 1997). A notable example is the *BARE-1* retrotransposon in the barley genome, which belongs to the *Copia*-like elements and is transcriptionally active. Copy number of *BARE-1* is positively correlated with factors such as genome size, temperature, water availability, soil type, altitude, and drought. The presence of ABA response elements in the *BARE-1* promoter region suggests that drought stress can trigger its propagation and induction (Suoniemi et al., 1996).

Aluminum (Al) toxicity in acidic soils has significant effects on plant growth and development and requires different responses in different plant species. The presence of genotypic differences in plant responses to aluminum has led to the development of different mechanisms for aluminum exclusion or tolerance. One important mechanism for Al tolerance is the efflux of organic anions (OAs) such as malate and citrate, which form stable complexes with *Al3+* (Tovkach et al., 2013). In plants exhibiting Al resistance, TEs were found in close proximity to or within OA transporter genes. While several of these TEs have been associated with enhanced OA efflux and increased Al resistance, some result in reduced resistance or show no significant effect (Sasaki et al., 2006; Collins et al., 2008). In wheat, the expression of genes involved in citrate and malate efflux plays a central role in Al resistance. The gene *TaMATE1B*, which is responsible for citrate transport, exhibits genotype-specific Al resistance due to the insertion of a transposon-like element upstream of its start codon. This insertion effectively extends the expression of *TaMATE1B* to the root tip, which increases Al tolerance(Tovkach et al., 2013). Interestingly, similar phenotypic results were observed in the context of citrate transporters in sorghum and barley, both of which exhibit increased Al resistance due to upstream TE insertions. These TEs, which belong to the class II TEs, include a small *MITE*-derived repeated region in the *SbMATE* gene of sorghum and a *CACTA* DNA-transposon that serves as a promoter for the *HvAACT1* gene of barley. These TE insertions have contributed significantly to the expansion of barley cultivation on acidic soils (Magalhaes et al., 2007).

Under iron stress conditions in the rice cultivar Nipponbare, the LTR-RTs show altered transcription, with most being up-regulated. A remarkable observation is that stress-resistant genes and LTR-RTs share common cis-regulatory elements, suggesting a possible interaction in their regulation under iron stress (Finatto et al., 2015). The insights gained by studying the complex interactions of TEs under stress conditions are promising for understanding the molecular basis of plant stress responses. Moreover, this knowledge could pave the way for the use of TEs as potential tools for innovative plant breeding strategies to improve stress tolerance and agricultural productivity (Paszkowski, 2015).

Previous studies show the dynamic and diverse involvement of TEs in plant stress responses “**Table 1**”. TEs contribute to the regulation of stress-responsive genes, generate phenotypic variation, and facilitate rapid adaptation to a changing environment. The interplay between TEs, epigenetic mechanisms, and phytohormones highlights the intricate molecular networks that control plant stress resistance and adaptation. Understanding the role and regulatory mechanisms of TEs in response to abiotic stress could pave the way for developing stress-tolerant crops and improving agricultural sustainability in the face of global environmental challenges.

**Table 1:** Some examples of the role of TEs in plant response to stress.

Plant species	Abiotic stress	Role of TEs in plant response to stress	Reference
Rice	Salinity, Frost	Induced insertion in specific site.	Naito et al. (2009)
Grapevine	Drought	Upregulation of gene expression in different tissues.	Ito et al. (2016)
Maize	Drought	Regulation of abiotic stress-responsive genes.	Makarevitch et al. (2015)
Various plants	Drought	Induced methylation and gene repression.	Galindo-González et al. (2018)
Z. mays	Drought	Differentially methylated regions linked to ABA responses.	Sallam and Moussa (2021)
Tomato	Drought, ABA	Activation by drought stress and ABA signaling pathways.	Benoit et al. (2019); Ito et al., 2016
Tomato, Tobacco	Freezing	Activation in response to cold temperatures.	Mhiri et al. (1997)
Barley	Drought	Activation in response to drought stress and ABA signaling.	Sallam and Moussa (2021)
Wheat, Barley	Aluminum	Change of organic anion efflux and resistance Al toxicity.	Delhaize et al. (2012)
Tomato	flooding	Increase the flooding tolerance.	Shiu et al. (1998)

## 6 TEs: tagging and mutagenesis in plant genomics

Transposon tagging is a powerful technique for identifying and studying mutant genes associated with specific traits, especially for understanding the function of unknown genes. This approach has been successfully applied to several species, including maize and other legumes. The technique involves insertional mutagenesis, which offers several advantages. First, it allows the precise identification of the genomic site where the transposon sequence is inserted using PCR-based methods, which facilitates the study of the affected genes. Second, the inserted sequences, called tags, can interact with neighboring genes, leading to various effects. These tags may contain elements that activate neighboring genes, enable the production of fusion proteins, or capture the expression patterns of nearby promoter sequences. These advanced tags serve as valuable resources for studying gene function, especially for essential and redundant genes that may not exhibit obvious loss-of-function phenotypes. The *bz1* gene in maize was the first target tagged with *Ac*. Since then, more than 60 genes related to basic plant development have been identified and cloned using transposons such as the suppressor-mutator (*Spm*) and mutator (*Mu*) elements from maize and the tag1 element from *Arabidopsis* (May and Martienssen, 2003). In addition, the enhancer/suppressor (En/Spm) element from maize has been used for mutagenesis in rice, extending the applicability of transposon tagging to other important plant species.

Transposon tagging is particularly valuable in polyploid or highly duplicated genomes such as wheat and soybean, where gene discovery is challenging due to the presence of multiple gene copies (Johnson et al., 2021). By disrupting the function of wild-type genes, transposons can be used to clone and sequence the affected genes. PCR is used to amplify the cloned fragments containing TEs and the gene of interest for further analysis. In addition, other mutagenesis techniques such as irradiation, chemical treatments, biological farming, arable farming, somaclonal variation, and double haploidy can be used to induce mutations in cells or tissues. Insertional mutagenesis uses different types of markers such as T-DNA, activator elements/dissociation elements (*Ac/Ds*), transposons, and retrotransposons. (Ram et al., 2019). Forward and reverse genetic approaches are being used to screen populations of insertional mutants in crops such as rice, and databases have been created for such populations. These populations serve as valuable resources for functional genomics studies in rice and have contributed to the elucidation of gene function.

Transposon-based insertional mutagenesis is proving to be a powerful tool for genetic improvement and molecular breeding in various plant species. The modified *mPing* element in rice serves as an activation marker that triggers nearby gene expression (Johnson et al., 2021). Bamboo’s active marine-like elements (MLEs), *Ppmar1* and *Ppmar2* with intact transposases, enable precise excisions and re-insertions, facilitating mutant libraries and gene tagging (Zhou et al., 2016). In addition, researchers examined the response of wild barley to tissue culture to optimize the introduction of *Ac/Ds* transposons by genetic transformation. It was found that the exogenous *Ac/Ds* elements were active in wild barley embryos/callus and resulted in transformation frequencies ranging from 72% to 100% in different wild barley genotypes, representing the first report of a transformation system in *H. vulgare* ssp. spontaneum (Cardinal et al., 2016). In *Arabidopsis thaliana*, transposon mutagenesis using the modified *Ac* element generates phenotypic mutants and facilitates specific labeling of loci. The tobacco retrotransposon *Tnt1* proves to be an effective insertional mutagen in potato, revealing phenotypic variation in plant growth and leaf morphology. In addition, the *Ac/Ds* system has been used in *Arabidopsis*, rice, and poplar to generate mutant populations that allow identification of mutant genomic regions by isolating flanking sequences of the *Ds* element (Kuromori et al., 2004). On the other hand, *PiggyBac* transposons have attracted attention due to their efficiency, safety, and stability in the transposition process. They can move relatively large DNA segments, ranging from 9.1 to 14.3 kb, and have shown promise for targeted gene insertion in plants. The architecture of *PiggyBac* transposons revealed by cryo EM reveals a synaptic complex and asymmetric dimer formation, with strand transfer and bending of target DNA characterized by unpaired TTAA sequences (Chen et al., 2020). Although *PiggyBac* transposons have been used for genome editing in plants, their use in plants is still limited compared with animals (Nishizawa-Yokoi et al., 2015). Transposon tagging and *PiggyBac* transposons offer exciting prospects for the advancement of plant breeding and functional genomics by providing valuable tools for deciphering gene function and improving plant traits. Moreover, the UniformMu transposon population was created to serve as a functional genomics tool for maize genetics. Systematic insertional mutagenesis of the maize genome was performed using the highly mutagenic native Robertson’s Mutator transposon system. McCarty et al. (2013) described the methods and protocols for genotyping of transposon insertion alleles.

## 7 TEs in genome editing

Transposition of TEs within a genome can have significant effects on neighboring genes and cause changes in genetic traits. This transposition can affect gene expression, splicing patterns, epigenetic changes, and overall gene functionality of genes located near the site where TE was inserted or removed. By manipulating regions adjacent to TEs or the TEs themselves through genome editing, it may be possible to replicate the natural translocation of TEs and potentially create new plants. One of the first TEs that has been used as a tool for genetic engineering is *Tnt1*, which has been successfully transformed into various plants such as tobacco, *Arabidopsis*, *Medicago truncatula*, and other legumes using different transformation methods, including leaf or cell culture protoplasts and *Agrobacterium* transformation (Orozco-Arias et al., 2019; Ramakrishnan et al., 2023). In addition, the *Tnt1* promoter can elicit a defense response in heterologous species, and treatments with CuCl2 and salicylic acid can generate transgenes that grant resistance to plant parasites and pathogens (Mhiri et al., 1997). Stimulation of *Tnt1* transposition by fungal extracts suggests a possible role in enhancing host genetic adaptability under environmental stress conditions (Melayah et al., 2001).

When a transgene is introduced into tobacco plants *via* the promoter of the *Tnt1* element, it is silenced, leading to an elevation of DNA methylation. Meanwhile, the endogenous *Tnt1* elements in tobacco remain partially methylated and undergo histone variant changes upon activation. This implies that the *Tnt1* promoter is the precise focal point of transcriptional gene silencing in tobacco plants (Hernández-Pinzón et al., 2012). Another example of LTR-TEs is the *Tto1* element used to produce transformed tobacco. The *Tto1* promoter plays a critical role in gene expression in transgenic plants that respond to tissue culture, wounding, fungal elicitors, and methyl jasmonate stimuli. Wounding and methyl jasmonate stimuli can induce *Tto1* in tobacco leaves (Takeda et al., 1999). Additionally, suppression of the retrotransposons *Tto1* and *Tar17* by tissue culture leads to a reduction in DNA methylation and gene silencing machinery in *Arabidopsis*. This reduction in methylation renders *Tto1* and *Tar17* transcriptionally active, demonstrating the role of DNA methylation and gene silencing machinery in transposon suppression (Hirochika et al., 2000). These results underscore the importance of understanding the intricate interplay between TEs and gene regulatory mechanisms in shaping plant genomes and responses to environmental cues.

In *Arabidopsis*, the LTR-RT named *OAR1* has been shown to confer increased tolerance to osmotic and alkaline stress in genetically modified plants. These transgenic plants show improved photochemical efficiency, intact membrane integrity, and enhanced expression of stress-responsive genes when exposed to osmotic and alkaline stress conditions, suggesting a positive role of *OAR1* in enhancing stress tolerance (Zhao et al., 2014). Another specific LTR-RT, Mikki, is highly expressed in the roots of rice and *Arabidopsis* plants during tissue culture. The transcripts of *Mikki* act as bait for a microRNA named miR171 and prevent the degradation of *SCARECROW -*Like mRNAs. As a result, *SCARECROW* -Like mRNAs specifically accumulate in the roots of different rice cultivars, indicating a possible role of retrotransposon-derived transcripts in tissue-specific regulation of gene expression (Cho and Paszkowski, 2017).

The *TLC1* family of LTR-RT from *Lycopersicon chilense* contains the necessary cis-regulatory elements for ethylene-induced gene expression in transgenic plants and protoplasts, suggesting that it is involved in the activation of stress-dependent gene transcription, particularly in response to ethylene signals (Tapia et al., 2005). In barley, the *BARE-1* LTR-RT has functional TATA boxes that are required for transient expression of introduced reporter genes in barley protoplasts. This suggests that it may serve as a retrotransposon for targeted epigenetic modifications and barley-specific propagation techniques (Vicient et al., 2001).

In *Saintpaulia* progeny obtained from tissue culture, the promoter region of the flavonoid 3’, 5’-hydroxylase (F3’5’H) gene remains consistent between different variants and individuals. Meanwhile, a transposon of the *hAT* superfamily was identified specifically in the promoter region of individuals of the variant. The flower color phenotypes observed are linked to the existence of insertions or deletions (indels) related to transposons. Excision of the *hAT* transposon affects the expression of F3’5’H, leading to variations in flower color (Sato et al., 2011). These examples highlight the multiple roles that LTR-RTs play in regulating gene expression and stress tolerance in different plant species, making them valuable targets for further research in plant improvement and stress resistance.

Introduction of the maize retrotransposon Δ*NaeAc* into flax callus by *Agrobacterium*-mediated transformation resulted in increased transcription and transposition within the callus, providing insights into the behavior of the *Ac* element in different plant species (Finnegan et al., 1993). In maize, the insertion of LTR retrotransposon *ZmRE-1* into the fifth exon of the brachytic2 (Br2) allele was found in dwarf mutants known as dwarf2014 (d2014), suggesting a possible link between *ZmRE-1* transposition and changes in plant height. This finding opens up possibilities for improving maize quality and yield through such transposition events (Li et al., 2020).

In *Lotus japonicus*, the retrotransposon *LORE1* can insert into highly repetitive sequences located in centromeres and telomeres, indicating its activity as a retrotransposon in intact plants and during *in vitro* tissue culture. The transposition of *LORE1* into gene-rich regions makes it an effective mutagenesis tool for studying gene function in plants (Madsen et al., 2005). Nevertheless, *LORE1* is epigenetically silenced in transgenic plants generated by *Agrobacterium*-mediated transformation, and the newly inserted *LORE1* elements do not show a strong preference for specific insertion sites. These unique features suggest the considerable potential of *LORE1* to induce genetic and epigenetic changes that contribute to evolutionary processes in host plants (Fukai et al., 2010).

The presence of the *ONSEN* retrotransposon in both *Arabidopsis* and *Raphanus sativus* suggests its ability to transpose into callus tissue exposed to heat stress during the regeneration process. This suggests a possible role of heat shock transcription factor and genes related to RNA-directed DNA methylation (RdDM) in regulating transcription and transposition of *ONSEN* when subjected to heat stress conditions. These results highlight the complex interplay between environmental factors and genetic elements in plants and reveal specific regulatory mechanisms involved in *ONSEN* retrotransposon activation and movement in response to heat stress (Masuta et al., 2017). These studies highlight the dynamic nature of retrotransposons and their potential importance in shaping genetic diversity and plant responses to various environmental stimuli.

The retrotransposon *EARE-1*, found in the genome of *Excoecaria agallocha*, shows increased expression in stressed organs, indicating the importance of horizontal transfers of retrotransposons and posttranscriptional gene silencing in the life cycle of *EARE-1* (Huang et al., 2017). In rice, mutant lines derived from callus culture of seed embryos contain newly generated copies of an active LTR-RT known as lullaby. This retrotransposon is thought to possess properties that contribute to the activation of transcription in rice callus, making it a promising candidate for a cis-acting element involved in this process. The discovery of Lullaby provides valuable insight into the mechanisms controlling transcriptional activation in rice callus and highlights its potential importance for understanding the molecular processes underlying callus culture and plant development (Picault et al., 2009).

*Tos17* has been effectively used by various methods to generate transgenic rice with novel traits. Its suitability for insertional mutagenesis and gene function analysis is due to its ability to proliferate under extended tissue culture time (Hirochika et al., 1996). In addition, *Tos17* has a prepossession for integration into gene regions, especially rapidly evolving gene classes, making it a valuable tool for studying gene function and its evolutionary dynamics in rice (Miyao et al., 2003). These examples demonstrate how retrotransposons can serve as powerful tools for understanding gene regulation, mutagenesis, and the molecular mechanisms of stress response and plant evolution.

The use of LTR-RT has brought several benefits, including increased genome stability, gene imprinting, incorporation of novel new gene functions, increased genetic variability, and improved stress tolerance in plants (Ramakrishnan et al., 2023). Using CRISPR/Cas9 genome editing technology, researchers were able to specifically mutagenize the *Tos17* retrotransposon in rice and successfully delete the region between LTRs in transformed calli. This breakthrough is an example of the successful removal of the *Tos17* retrotransposon from the rice genome by CRISPR/Cas9-mediated targeted mutagenesis and opens new opportunities for the study of gene function and epigenetics (Saika et al., 2019). In addition, CRISPR/Cas9-mediated gene editing led to the creation of the *Tos17* D873 allele in rice, suggesting that specific domains of *Tos17* are critical for transposition. This innovation holds the potential to generate transgenic rice plants for the study of gene function and epigenetic (Luo et al., 2020). Similarly, fusion of the *ATCOPIA93* retrotransposon promoter with a GUS gene in *Arabidopsis* has shown immune-responsive behavior, suggesting a plausible link between retrotransposons and plant response to biotic stress. This finding opens up possibilities for targeting LTR-RTs to enhance plant immune responses to pathogens (Zervudacki et al., 2018).

Furthermore, the occurrence of numerous copies of the HUO LTR-RT in rice resulting from reciprocal crosses could potentially trigger genomic instability, influencing the production of small RNAs and genome-wide DNA methylation. In addition, the presence of multiple copies of the *HUO* LTR-RT in rice from reciprocal crosses may lead to genomic instability that affects the production of small RNAs and genome-wide DNA methylation. Such instability could negatively impact disease resistance and crop yield, highlighting the importance of retrotransposon control in rice breeding programs to improve productivity and disease resistance (Park et al., 2014). In addition, the CLEVR technique enabled monitoring of *Gypsy* LTR-RT replication in cell cultures and neurons, where disruption of the siRNA pathway resulted in increased replication rates. This research provides valuable insight into the dynamics of retrotransposon replication and its potential effects in different organisms and provides important clues for future studies (Chang et al., 2019).

Overall, the integration of LTR-RTs as a study object has provided valuable insights and promising prospects for improving plant genetics, enhancing plant traits, and deciphering the intricacies of gene regulation. These results highlight the central role of retrotransposons in plant genome design and provide exciting opportunities for further advances in crop improvement and understanding gene expression and function.

## 8 LTR-RTs in genetic engineering: challenges and advancements

In the field of plant genetic engineering, the utilization of LTR-RTs poses both highlights and challenges. A key challenge lies in the difficulty of controlling copy numbers and variations in copy expression. Retrotransposons can exist in high quantities, ranging from hundreds to hundreds of thousands of copies, which can exert both positive and negative impacts on gene functions and genome stability. To counter this, plants have developed intricate regulatory mechanisms, such as epigenetic modifications and DNA methylation, to suppress retrotransposon activity and maintain copy number equilibrium. Nonetheless, the precise mechanisms governing copy number regulation remain largely unexplored. The interplay between epigenetic modifications and retrotransposon silencing is vital for safeguarding genome integrity (Ramakrishnan et al., 2023).

Controlling retrotransposons presents several challenges due to variations in copy number expression within the same family, species, and developmental stages, alongside insertion site heterogeneity and polymorphism. Measuring and controlling copy numbers accurately is arduous, particularly since retrotransposons give rise to multiple genes and noncoding RNAs. Repression of retrotransposons is influenced by the activity of host RNA polymerase II, which has a pivotal role in their mobility. Additionally, a combination of DNA methylation inhibition and *Pol II* activity can induce stress-dependent mobilization of specific retrotransposons in plants, exemplified by *ONSEN* in *Arabidopsis* (Thieme et al., 2017). Throughout different stages of plant growth and development, epigenetic changes play a vital role in the activation and deactivation of multiple retrotransposon copies. For instance, in *Arabidopsis*, a mutation in the maintenance methyltransferase 1 (*MET1*) gene triggers the activation of *EVADÉ*, an *ATCOPIA93* retrotransposon, specifically during sexual reproduction, leading to retrotransposon amplification (Mirouze et al., 2009).

To address the challenges of employing LTR-RTs in plant genome editing, further technological advancement is required. Advanced technology, such as next-generation sequencing and CRISPR-Cas9, has significantly impacted the use of TEs as genetic engineering tools. Advanced sequencing has offered deeper insights into transposons, their diversity, and distribution across different genomes. Meanwhile, CRISPR-Cas9 technology enables precise targeting and manipulation of transposons within the genome. Machine learning algorithms have the potential to automatically learn parameters and develop models for specific retrotransposon-related issues. Recent progress in computational and third-generation sequencing technologies provides improved methods for understanding retrotransposon movement, gene silencing/activation, copy number variation, and expression, which will enhance the control of LTR-RTs in the future. The application of advanced technology has opened new possibilities in various fields, including agriculture, medicine, and biotechnology, expanding the potential of transposon elements (Ramakrishnan et al., 2023).

## 9 LncRNAs: impact of TEs on gene regulation

When present in RNA transcripts, transposable elements have been shown to significantly impact gene regulation. Whether present in long noncoding RNAs (lncRNAs) or messenger RNAs (mRNAs), these elements can affect the processing, stability, and localization of these transcripts (Fort et al., 2021). LncRNAs are RNA molecules longer than 200 nucleotides that have been shown to be important players in regulating gene expression (Qin et al., 2017). They are found in various plant species and originate from intronic, exonic, and intergenic regions. The number of lncRNAs varies among species and ranges from thousands to tens of thousands. For instance, 2986 and 18,031 lncRNAs have been reported for *Arabidopsis* and maize, whereas for species such as *Arabidopsis lyrata*, rice, and poplar, 400-6000 lncRNAs have been identified. Interestingly, there is a positive correlation between the abundance of lncRNAs and the number of protein-coding genes in these species, reflecting their potential importance in gene regulation (Deng et al., 2017).

LncRNAs can be classified into several classes based on their genomic location and relationship to neighboring protein-coding genes. These classes include intergenic lncRNAs (located between genes), intronic lncRNAs (derived from introns of proteincoding genes), sense lncRNAs (overlapping with protein-coding genes on the same strand), antisense lncRNAs (that overlap with protein-coding genes on the opposite strand), and bidirectional lncRNAs (that are transcribed in the opposite direction from nearby protein-coding genes). These classifications contribute to a better understanding of the diversity and functional properties of lncRNAs (Statello et al., 2021). Despite sequence divergence among plant species, relative position conservation is observed for lncRNAs, and lncRNA exons share conserved splicing signals among themselves and with protein-coding genes, such as “GTAG” (Li et al., 2014). Most lncRNAs in plants are transcribed by RNA polymerase II and undergo posttranscriptional modifications, including 5’-capping, polyadenylation, and splicing. However, a small number of lncRNAs are transcribed by RNA polymerase III. The plant-specific RNA polymerases IV and V were also found to transcribe lncRNAs. In particular, RNA polymerase V plays a role in the RNA-directed DNA methylation (RdDM) pathway, suggesting that it is involved in the regulation of gene expression by DNA methylation (Fort et al., 2021; Statello et al., 2021).

TE-derived long noncoding RNAs (TE-lncRNAs) can serve as precursors for microRNAs (miRNAs) and small interfering RNAs (siRNAs). The intricate regulatory networks formed by these non-coding RNAs interact with their target genes, resulting in the control of multiple genes that ultimately determine the overall plant response to environmental stress and pathogen invasion (Gelaw and Sanan-Mishra, 2021). The study of lncRNAs and their interactions with TEs provides valuable insights into the complexity of gene regulatory mechanisms in plants. It has been shown that lncRNAs are essential regulators in several physiological processes, including fertility, nutrient homeostasis, nodulation, metabolic pathways, axillary root growth, and modulation of signal transduction of plant growth hormones such as auxin, gibberellin (GA), cytokinin, and ethylene (Fort et al., 2021). In addition, lncRNAs play an important role in secondary wood formation in economically important plant species. Similar to TEs, lncRNAs are also involved in modulating chromosome structure, regulating transcriptional splicing, maintaining mRNA stability, and modifying post-translation processes, underscoring their versatile functions in plant biology (Fort et al., 2021). In addition, lncRNAs play a role in chromatin remodeling and histone modification during various developmental stages, including reproduction, embryogenesis, and organogenesis, especially in response to stress conditions. For example, the cold-induced lncRNA COOLAIR represses FLC (Flowering Locus C) expression during vernalization in *Arabidopsis*, thereby controlling the timing of flowering. This repression mechanism involves the recruitment of the Polycomb repressive complex 2 (PRC2) and the addition of trimethyl groups to histone H3 at lysine 27 (H3K27me3) at the FLC gene locus (Gelaw and Sanan-Mishra, 2021). LncRNAs also shape the distinct properties of promoters of coding genes and are subject to regulation by various transcription factors, including p53, nuclear factor kappa B (NF-KB), Sox-2, and Oct 4 (Guttman et al., 2009).

Noncoding RNAs, particularly long intergenic noncoding RNAs (lincRNAs), have gained interest in the study of plant responses to abiotic stresses (Jha et al., 2020). TEs associated with lincRNAs (TE-lincRNAs) exhibit stress-induced expression patterns in *Arabidopsis*, maize, and rice, suggesting that they are involved in stress-responsive transcription (Wang et al., 2012). The expression of TE-lincRNAs is affected by various stresses such as salinity, cold, and ABA. In addition, *Arabidopsis* mutants lacking TE-lincRNAs showed reduced sensitivity to ABA, suggesting a role in the ABA-abiotic stress response (Wang et al., 2017). Recent evidence also suggests that certain lincRNAs control drought-responsive genes, including ABA-signaling genes, which is an exciting area for further research (Jha et al., 2020).

## 10 TEs as Molecular markers

Retrotransposable elements are widespread in eukaryotic genomes, and their mobility by reverse RNA transcription can lead to these elements being inserted at new sites. During meiotic prophase, a phase of cell division, these repeat elements may undergo internal rearrangements, and their copy number may fluctuate. Among retro transposons, LTR-RTs, characterized by their LTR, exhibit random insertion patterns that may contribute to species evolution. These elements are valuable tools for study9 ing evolutionary processes, species differences, and genome variations (Kalendar and Schulman, 2006; Ratet et al., 2010; Kalendar et al., 2019). Retrotransposon-based DNA markers derived from LTR-RTs play a critical role in the study of genetic diversity and variation (Vuorinen et al., 2018; Ghonaim et al., 2020). Such markers are widely used to generate genetic maps and identify individuals or lines with specific genetic variations, which is helpful in molecular breeding and crop improvement (Khapilina et al., 2021). In addition, these molecular markers enable the study of genetic diversity in crops and retrotransposon activation under abiotic stress conditions (Kalendar and Schulman, 2006; Belyayev et al., 2010). LTR-RTs are also linked to key genes that serve as targets for genome assembly, variation analysis, gene tagging, and the study of functional genes, making them valuable resources for molecular breeding strategies (Potter, 2005). The Inter Retrotransposon Amplified Polymorphism (IRAP) method is a powerful tool for identifying retrotransposon insertion polymorphisms (Kalendar et al., 1999). It allows amplification of DNA regions between two retroelements using outward primers from LTR sequences. Different orientations of retrotransposons, such as head-to-head, tail-to-tail, or head-to-tail, affect genetic diversity and facilitate species-specific identification (Poczai et al., 2013). The IRAP method has been successfully used to study genetic diversity in various plant species such as sunflower, pine, sugarcane, *Xanthosoma* and *Colocasia* (Vukich et al., 2009; Fan et al., 2014; Doungous et al., 2015; Shingote et al., 2019) **Table 2**.

**Table 2:** List of various examples of TEs-based molecular markers and their applications.

Molecular Marker	Plant species	Application	References
IRAP	*Helianthus species*	Genetic diversity	Vukich et al. (2009)
IRAP	Pine species	Genetic diversity	Fan et al. (2014)
IRAP	Sugarcane	Genetic integrity	Shingote et al. (2019)
IRAP	Xanthosoma and Colocasia	Intraspecific variability	Doungous et al. (2015)
SSAP	Vitis genus	Study *Copia* family	Moisy et al. (2008)
SSAP	Tetraploid Wheat	TEs and defense mechanisms	Woodrow et al. (2010)
SSAP	Allotetraploid Tobacco	Genetic analysis	Petit et al. (2010)
SSAP	Cashew and Myrtle	Genetic polymorphism	Woodrow et al. (2012); Syed et al. (2005)
REMAP and IRAP	Rice and Barely	Genetic diversity	Branco et al. (2007)
REMAP	Lentil	Lentil breeding	Rey-Baños et al. (2017)
IRAP and REMAP	Cassava cultivars	Genetic diversity	Kuhn et al. (2016)
RBIP	Sweet potatoes	Genetic diversity	Meng et al. (2021)
RBIP	Asian rice	Origin of Asian rice cultivars	Roy et al. (2015)
RBIP	Pears	Pears breeding	Kim et al. (2012)
RBIP	Melilotus	Genetic improvement	Ouyang et al. (2021)
RBIP	*Pisum sativum*	Genetic diversity and evaluation	Jing et al. (2010)
IRAP and RBIP	Potato cultivars	Genetic diversity	Sharma and Nandineni (2014)
IRAP	*Lilium* species	Genetic diversity	Lee et al. (2015)
iPBS	Plants and Animals	Genetic diversity	Kalendar et al. (2010)
iPBS	*Gnetum* species	Molecular characterization	Doungous et al. (2020)
RJJMs	*Aegilops tauschii*	Genome mapping	You et al. (2010)
PST	Timothy-grass	Marker development	Kalendar et al. (2019)

The Sequence-Specific Amplification Polymorphism (SSAP) technique is a modified Amplified Fragment Length Polymorphism (AFLP) protocol that uses prior knowledge of transposon sequences and a single restriction digest to amplify transposon insertion sites in genomic DNA (Syed and Flavell, 2007). In the SSAP method, genomic DNA is first digested with a restriction enzyme, followed by the addition of adaptors to the restrictive ends. PCR is then performed with primers specific for the adaptors and the 3’ end of the LTR sequences of the transposons (Poczai et al., 2013). The resulting amplicons represent the transposon insertion sites and can be used to study transposon activity and the effects of genomic perturbations, such as polyploidization, on transposon mobilization. For example, SSAP has been used to study ten *Copia*-like retrotransposon families in the genus *Vitis* (Moisy et al., 2008). In addition, SSAP has played a critical role in revealing the importance of *Copia*-like retrotransposon elements, particularly their retrotransposition, in the defense mechanisms of tetraploid wheat in response to environmental stress (Woodrow et al., 2010).

The SSAP method has been used for genetic analysis of synthetic allotetraploid tobacco (Petit et al., 2010). In addition, methylation-sensitive version of SSAP was used to study epigenetic changes near retrotransposons in hybrid and allopolyploid *Spartina* genomes (Parisod et al., 2009). Parisod et al. (2009) showed that these changes occurred rapidly and were more pronounced in the maternal subgenome, suggesting that the environment of TEs was significantly affected by methylation changes specific to the maternal genome. In cashew and myrtle genomes, LTR-RT-based SSAP markers had a significantly higher proportion of polymorphic markers compared to AFLP markers, increasing their utility for genetic studies (Syed et al., 2005; Woodrow et al., 2012).

The REMAP (REtrotransposon-Microsatellite Amplified Polymorphism) protocol is a valuable method for distinguishing different genotypes within a species using microsatellites or short and highly redundant sequence repeats with LTR-specific primers in PCR (Kalendar and Schulman, 2006; Kalendar et al., 2021). Similar to the IRAP method, REMAP can be used in combination to distinguish species within a genus and has been used to distinguish barley and rice varieties (Kalendar et al., 1999; Branco et al., 2007). In lentils, retrotransposons are not randomly distributed across the genome, with marker density varying in different regions, making REMAP potentially useful for marker-assisted lentil breeding (Rey-Baños et al., 2017). The IRAP and REMAP markers in cassava plants exhibit a high degree of polymorphism, mak1 ing them useful tools for studying genetic diversity and relationships among different cassava cultivars (Kuhn et al., 2016). The retrotransposon-based insertion polymorphism (RBIP) technique uses three primers to detect polymorphism in retrotransposon insertions. The result is codominant markers that provide valuable phylogenetic information and help protect plant breeders’ rights. RBIP has been used to study the origin of Asian rice varieties and generate markers for marker-assisted breeding of pears (Kim et al., 2012; Roy et al., 2015). Genome-wide analysis of RBIP markers in Melilotus genomes revealed high polymorphism information content (PIC), making them valuable tools for genetic improvement of the genus Melilotus (Ouyang et al., 2021).

The combination of IRAP and RBIP markers has also shown high polymorphism in several retrotransposon families, providing valuable insights into genetic diversity among different potato cultivars (Sharma and Nandineni, 2014). These methods have also been used to determine genetic diversity in *Lilium* species (Lee et al., 2015), grasses such as *Cleistogenes songorica* and strawberry, and chokeberry, which has improved our understanding of genetic variation, QTL mapping, population structure, and germplasm evolution (Monden et al., 2014; Liang et al., 2016; Ma et al., 2021). In pepper, species-specific retrotransposon-based markers have been developed to detect polymorphism of *Capsicum annuum* species, which are valuable tools for genetic studies (Yañez-Santos et al., 2021). The iPBS amplification marker targets the common presence of a tRNA complement as a reverse transcriptase primer binding site (PBS) in LTR-retrotransposons and allows broader application to different retrotransposon types (Khapilina et al., 2021). Other innovative approaches, such as Palindromic Sequence-Targeted (PST) PCR, which combines a palindromic 6 bp sequence (PST site) and conserved TE sequences, enable genome walking, profiling, and assessment of genetic variability within and between species (Kalendar et al., 2019). Molecular markers derived from class II DNA transposons such as *MITE* and *CACTA* have been used for phylogenetic and genetic analyses in *Arabidopsis* and rice species due to their unique properties (Kwon et al., 2006; Park et al., 2014). TEMM is a curated database that contains the TE-based molecular markers mentioned above as well as their corresponding primer sequences and PCR conditions (https://bioinformatics.um6p.ma/TEMM) (Hassan et al., 2023). In summary, these various retrotransposon-based marker techniques are valuable tools for studying genetic diversity, evolutionary relationships, and marker-assisted breeding in various plant species. Their ability to target specific retrotransposon elements and generate codominant markers improves our understanding of genetic variation and enables effective management of plant genetic resources.

## 11 Conclusion

TEs are essential components of eukaryotic genomes that play a variety of roles in gene regulation, genome evolution, and environmental adaptation. Their diverse functions, from gene regulation to biotechnological tools, make them invaluable resources for genetic and evolutionary studies. Continued research on TEs, along with advances in biotechnology, offers promising opportunities to address agricultural challenges, understand genome dynamics, and develop sustainable crop management strategies in the face of changing environmental conditions. Harnessing the potential of TEs will pave the way for exciting developments in plant genetics and biotechnology that will benefit agriculture and food security on a global scale. Understanding the mobility, regulation, and interactions of TE with other genomic elements is critical to using them as molecular markers and advancing genetic studies. Their occurrence in plant genomes provides insights into genetic diversity and speciation within species. Further exploration of the mechanisms controlling the mobility of TE, epigenetic regulation, and interactions with other genomic elements is essential. Deciphering the complexity of TE behavior will provide a more comprehensive understanding of its impact on genome evolution and gene regulation. In the field of plant biology, the use of TEs as molecular markers may lead to improved breeding strategies, increased genetic diversity, and the development of stress-resistant varieties. In addition, the use of TEs for targeted mutagenesis and gene tagging opens exciting possibilities for functional genomics and the discovery of genes with complex genetic backgrounds.

## Author contributions

AH: Methodology, Investigation, Writing – original draft. MM: Conceptualization, Methodology, Writing – review & editing. AA: Methodology, Conceptualization, Supervision, Writing – review & editing.
